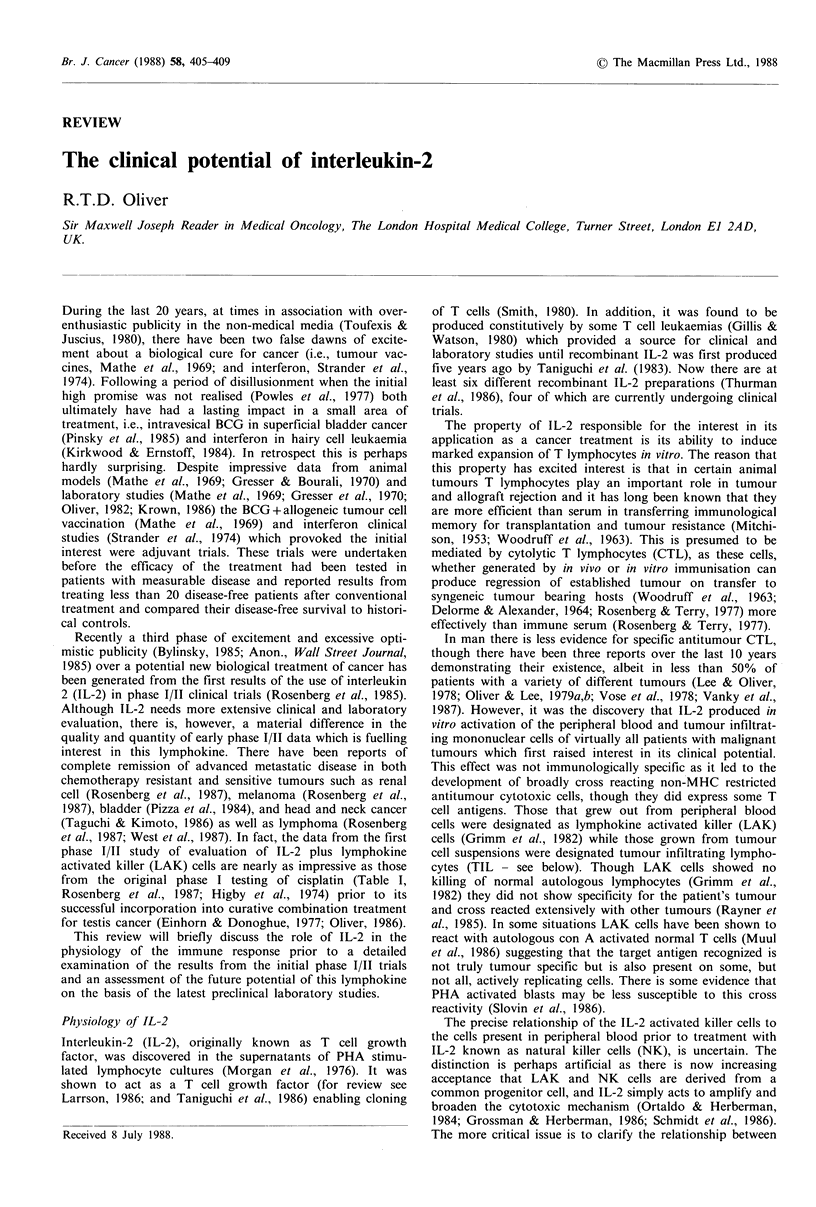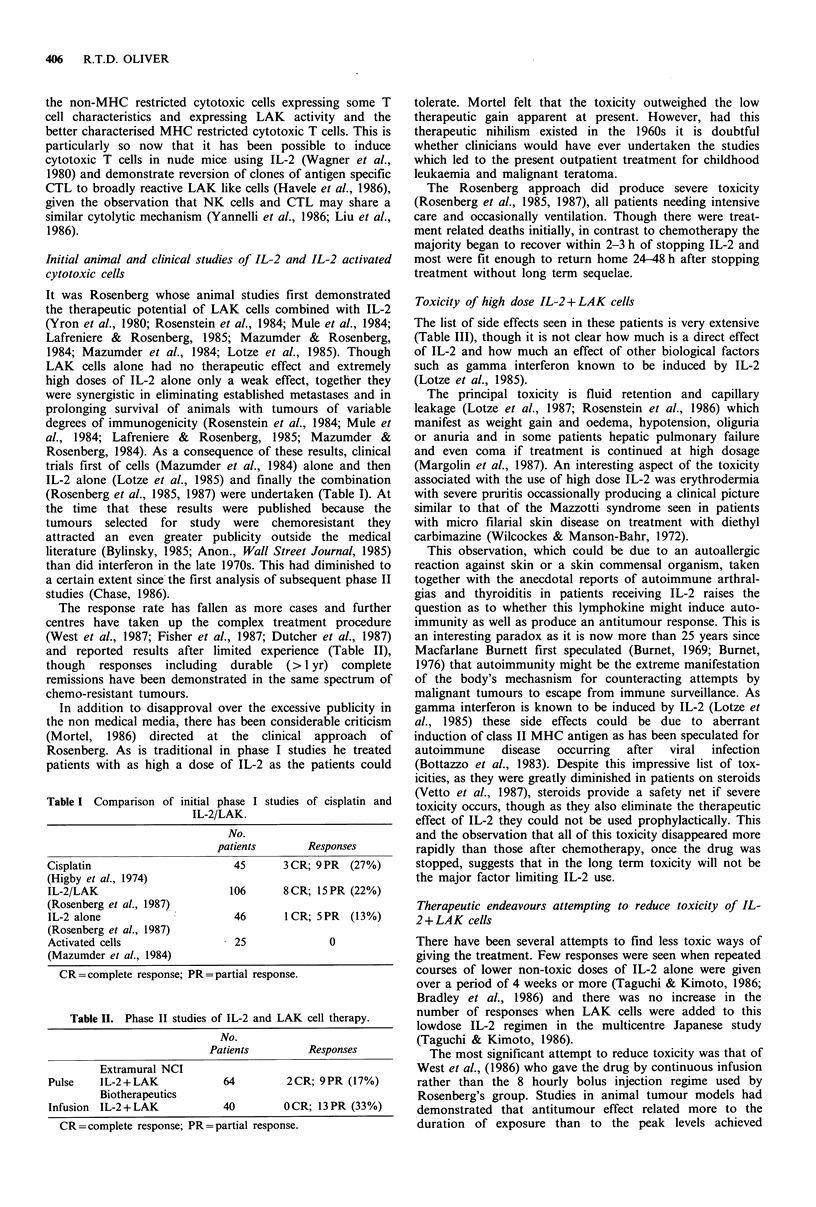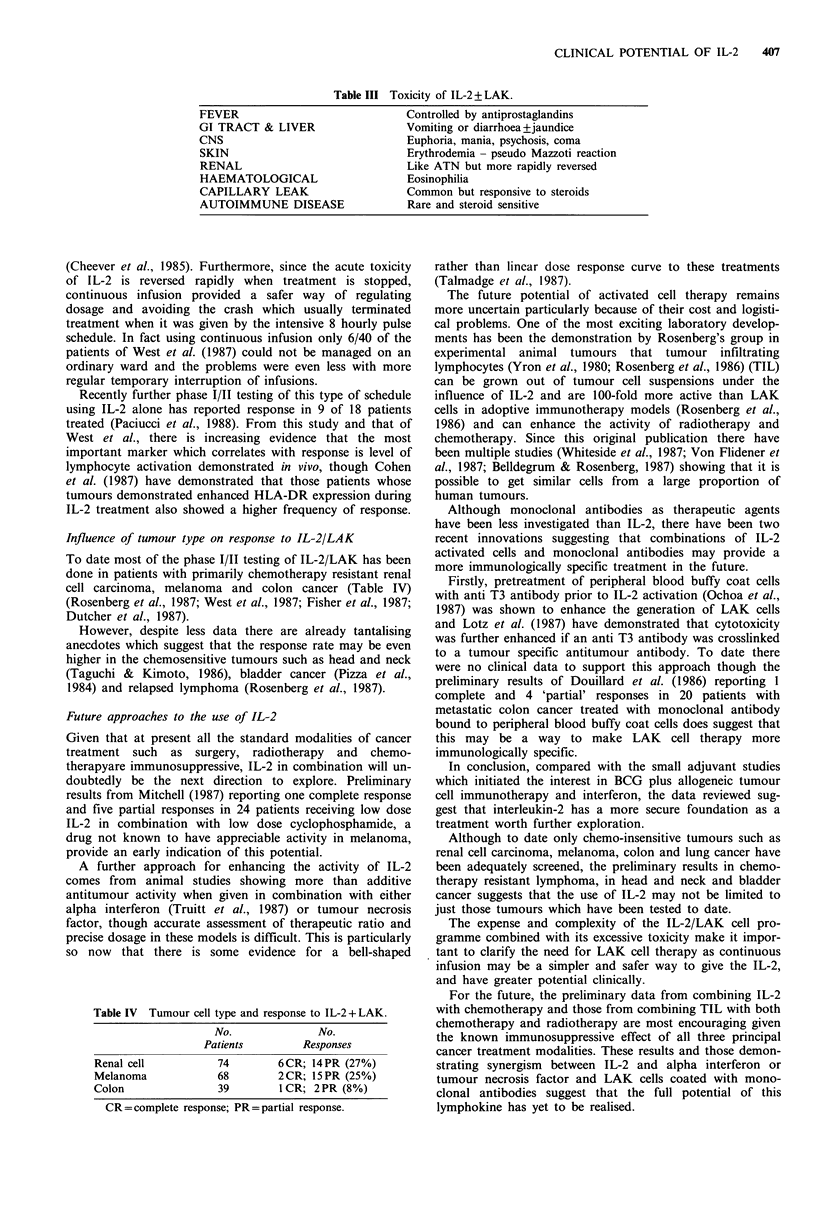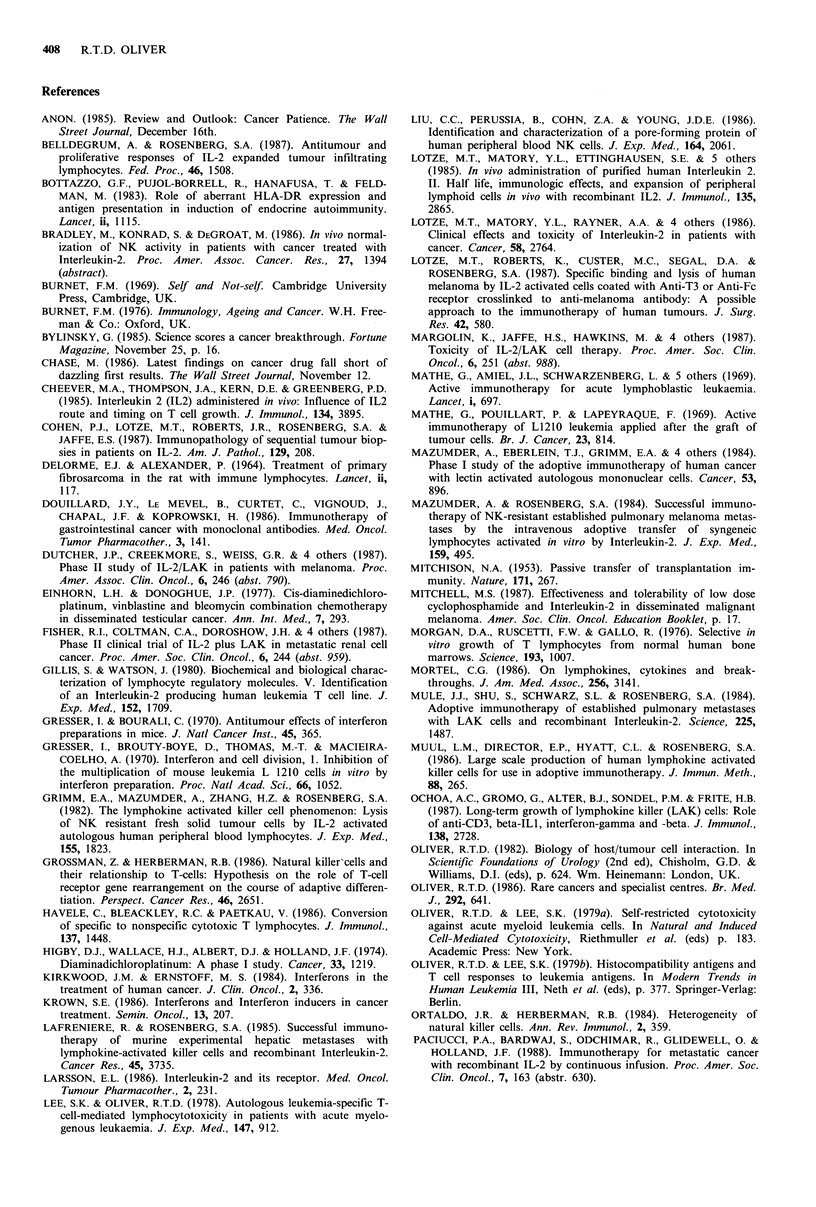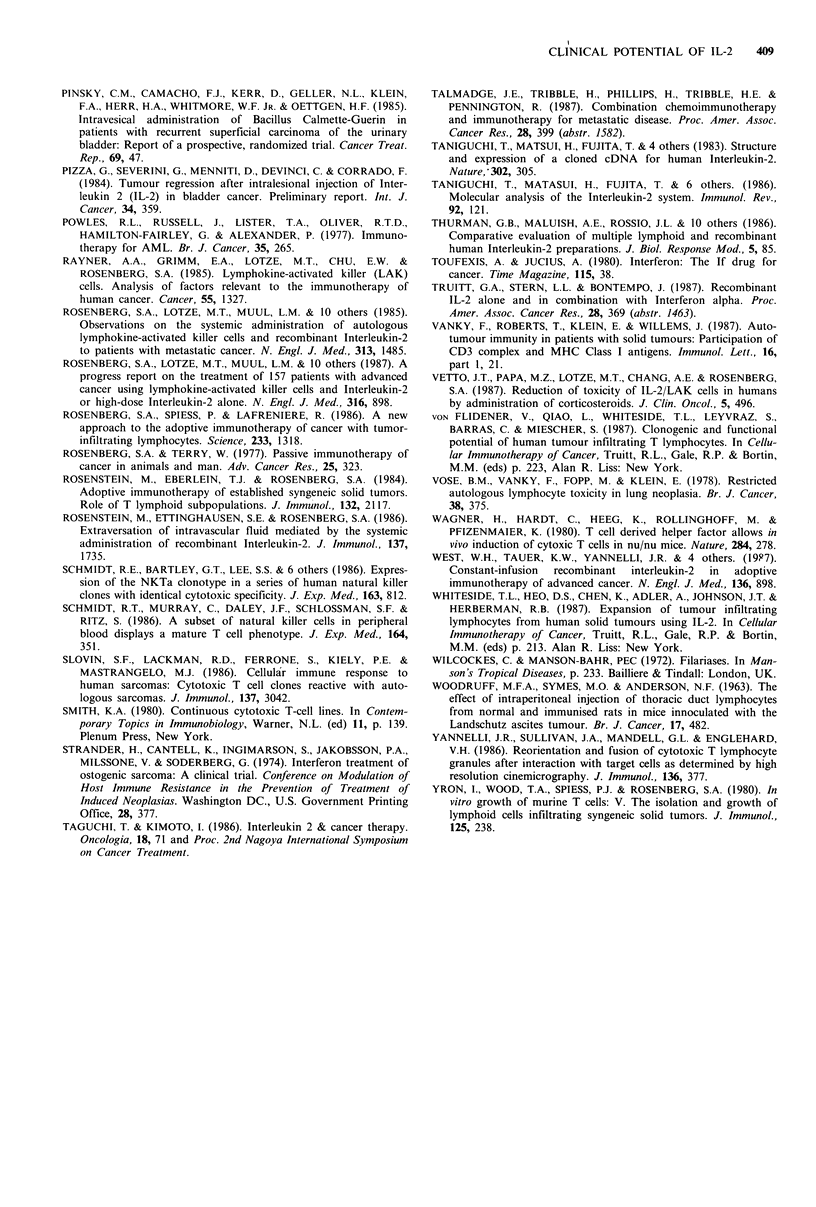# The clinical potential of interleukin-2.

**DOI:** 10.1038/bjc.1988.230

**Published:** 1988-10

**Authors:** R. T. Oliver

**Affiliations:** London Hospital Medical College, UK.


*Br. J. Cancer* (1988) **58**, 405–409 © The Macmillan Press Ltd., 1988

**REVIEW**

### The clinical potential of interleukin-2

R.T.D. Oliver

*Sir Maxwell Joseph Reader in Medical Oncology, The London Hospital Medical College, Turner Street, London E1 2AD, UK.*

During the last 20 years, at times in association with over-enthusiastic publicity in the non-medical media (Toufexis & Juscius, 1980), there have been two false dawns of excitement about a biological cure for cancer (i.e., tumour vaccines, Mathe *et al.*, 1969; and interferon, Strander *et al.*, 1974). Following a period of disillusionment when the initial high promise was not realised (Powles *et al.*, 1977) both ultimately have had a lasting impact in a small area of treatment, i.e., intravesical BCG in superficial bladder cancer (Pinsky *et al.*, 1985) and interferon in hairy cell leukaemia (Kirkwood & Ernstoff, 1984). In retrospect this is perhaps hardly surprising. Despite impressive data from animal models (Mathe *et al.*, 1969; Gresser & Bourali, 1970) and laboratory studies (Mathe *et al.*, 1969; Gresser *et al.*, 1970; Oliver, 1982; Krown, 1986) the BCG + allogeneic tumour cell vaccination (Mathe *et al.*, 1969) and interferon clinical studies (Strander *et al.*, 1974) which provoked the initial interest were adjuvant trials. These trials were undertaken before the efficacy of the treatment had been tested in patients with measurable disease and reported results from treating less than 20 disease-free patients after conventional treatment and compared their disease-free survival to historical controls.

Recently a third phase of excitement and excessive optimistic publicity (Bylinsky, 1985; Anon., *Wall Street Journal*, 1985) over a potential new biological treatment of cancer has been generated from the first results of the use of interleukin 2 (IL-2) in phase I/II clinical trials (Rosenberg *et al.*, 1985). Although IL-2 needs more extensive clinical and laboratory evaluation, there is, however, a material difference in the quality and quantity of early phase I/II data which is fuelling interest in this lymphokine. There have been reports of complete remission of advanced metastatic disease in both chemotherapy resistant and sensitive tumours such as renal cell (Rosenberg *et al.*, 1987), melanoma (Rosenberg *et al.*, 1987), bladder (Pizza *et al.*, 1984), and head and neck cancer (Taguchi & Kimoto, 1986) as well as lymphoma (Rosenberg *et al.*, 1987; West *et al.*, 1987). In fact, the data from the first phase I/II study of evaluation of IL-2 plus lymphokine activated killer (LAK) cells are nearly as impressive as those from the original phase I testing of cisplatin (Table I, Rosenberg *et al.*, 1987; Higby *et al.*, 1974) prior to its successful incorporation into curative combination treatment for testis cancer (Einhorn & Donoghue, 1977; Oliver, 1986).

This review will briefly discuss the role of IL-2 in the physiology of the immune response prior to a detailed examination of the results from the initial phase I/II trials and an assessment of the future potential of this lymphokine on the basis of the latest preclinical laboratory studies.

### *Physiology of IL-2*

Interleukin-2 (IL-2), originally known as T cell growth factor, was discovered in the supernatants of PHA stimulated lymphocyte cultures (Morgan *et al.*, 1976). It was shown to act as a T cell growth factor (for review see Larrson, 1986; and Taniguchi *et al.*, 1986) enabling cloning of T cells (Smith, 1980). In addition, it was found to be produced constitutively by some T cell leukaemias (Gillis & Watson, 1980) which provided a source for clinical and laboratory studies until recombinant IL-2 was first produced five years ago by Taniguchi *et al.* (1983). Now there are at least six different recombinant IL-2 preparations (Thurman *et al.*, 1986), four of which are currently undergoing clinical trials.

The property of IL-2 responsible for the interest in its application as a cancer treatment is its ability to induce marked expansion of T lymphocytes *in vitro*. The reason that this property has excited interest is that in certain animal tumours T lymphocytes play an important role in tumour and allograft rejection and it has long been known that they are more efficient than serum in transferring immunological memory for transplantation and tumour resistance (Mitchison, 1953; Woodruff *et al.*, 1963). This is presumed to be mediated by cytolytic T lymphocytes (CTL), as these cells, whether generated by *in vivo* or *in vitro* immunisation can produce regression of established tumour on transfer to syngeneic tumour bearing hosts (Woodruff *et al.*, 1963; Delorme & Alexander, 1964; Rosenberg & Terry, 1977) more effectively than immune serum (Rosenberg & Terry, 1977).

In man there is less evidence for specific antitumour CTL, though there have been three reports over the last 10 years demonstrating their existence, albeit in less than 50% of patients with a variety of different tumours (Lee & Oliver, 1978; Oliver & Lee, 1979*a,b*; Vose *et al.*, 1978; Vanky *et al.*, 1987). However, it was the discovery that IL-2 produced *in vitro* activation of the peripheral blood and tumour infiltrating mononuclear cells of virtually all patients with malignant tumours which first raised interest in its clinical potential. This effect was not immunologically specific as it led to the development of broadly cross reacting non-MHC restricted antitumour cytotoxic cells, though they did express some T cell antigens. Those that grew out from peripheral blood cells were designated as lymphokine activated killer (LAK) cells (Grimm *et al.*, 1982) while those grown from tumour cell suspensions were designated tumour infiltrating lymphocytes (TIL – see below). Though LAK cells showed no killing of normal autologous lymphocytes (Grimm *et al.*, 1982) they did not show specificity for the patient's tumour and cross reacted extensively with other tumours (Rayner *et al.*, 1985). In some situations LAK cells have been shown to react with autologous con A activated normal T cells (Muul *et al.*, 1986) suggesting that the target antigen recognized is not truly tumour specific but is also present on some, but not all, actively replicating cells. There is some evidence that PHA activated blasts may be less susceptible to this cross reactivity (Slovin *et al.*, 1986).

The precise relationship of the IL-2 activated killer cells to the cells present in peripheral blood prior to treatment with IL-2 known as natural killer cells (NK), is uncertain. The distinction is perhaps artificial as there is now increasing acceptance that LAK and NK cells are derived from a common progenitor cell, and IL-2 simply acts to amplify and broaden the cytotoxic mechanism (Ortaldo & Herberman, 1984; Grossman & Herberman, 1986; Schmidt *et al.*, 1986). The more critical issue is to clarify the relationship between

Received 8 July 1988.

the non-MHC restricted cytotoxic cells expressing some T cell characteristics and expressing LAK activity and the better characterised MHC restricted cytotoxic T cells. This is particularly so now that it has been possible to induce cytotoxic T cells in nude mice using IL-2 (Wagner *et al.*, 1980) and demonstrate reversion of clones of antigen specific CTL to broadly reactive LAK like cells (Havele *et al.*, 1986), given the observation that NK cells and CTL may share a similar cytolytic mechanism (Yannelli *et al.*, 1986; Liu *et al.*, 1986).

*Initial animal and clinical studies of IL-2 and IL-2 activated cytotoxic cells*

It was Rosenberg whose animal studies first demonstrated the therapeutic potential of LAK cells combined with IL-2 (Yron *et al.*, 1980; Rosenstein *et al.*, 1984; Mule *et al.*, 1984; Lafreniere & Rosenberg, 1985; Mazumder & Rosenberg, 1984; Mazumder *et al.*, 1984; Lotze *et al.*, 1985). Though LAK cells alone had no therapeutic effect and extremely high doses of IL-2 alone only a weak effect, together they were synergistic in eliminating established metastases and in prolonging survival of animals with tumours of variable degrees of immunogenicity (Rosenstein *et al.*, 1984; Mule *et al.*, 1984; Lafreniere & Rosenberg, 1985; Mazumder & Rosenberg, 1984). As a consequence of these results, clinical trials first of cells (Mazumder *et al.*, 1984) alone and then IL-2 alone (Lotze *et al.*, 1985) and finally the combination (Rosenberg *et al.*, 1985, 1987) were undertaken (Table I). At the time that these results were published because the tumours selected for study were chemoresistant they attracted an even greater publicity outside the medical literature (Bylinsky, 1985; Anon., *Wall Street Journal*, 1985) than did interferon in the late 1970s. This had diminished to a certain extent since the first analysis of subsequent phase II studies (Chase, 1986).

The response rate has fallen as more cases and further centres have taken up the complex treatment procedure (West *et al.*, 1987; Fisher *et al.*, 1987; Dutcher *et al.*, 1987) and reported results after limited experience (Table II), though responses including durable (>1 yr) complete remissions have been demonstrated in the same spectrum of chemo-resistant tumours.

In addition to disapproval over the excessive publicity in the non medical media, there has been considerable criticism (Mortel, 1986) directed at the clinical approach of Rosenberg. As is traditional in phase I studies he treated patients with as high a dose of IL-2 as the patients could tolerate. Mortel felt that the toxicity outweighed the low therapeutic gain apparent at present. However, had this therapeutic nihilism existed in the 1960s it is doubtful whether clinicians would have ever undertaken the studies which led to the present outpatient treatment for childhood leukaemia and malignant teratoma.

**Table I** Comparison of initial phase I studies of cisplatin and IL-2/LAK.

| | *No. patients* | *Responses* |
|---|---|---|
| Cisplatin (Higby *et al.*, 1974) | 45 | 3 CR; 9 PR (27%) |
| IL-2/LAK (Rosenberg *et al.*, 1987) | 106 | 8 CR; 15 PR (22%) |
| IL-2 alone (Rosenberg *et al.*, 1987) | 46 | 1 CR; 5 PR (13%) |
| Activated cells (Mazumder *et al.*, 1984) | 25 | 0 |

CR = complete response; PR = partial response.

**Table II.** Phase II studies of IL-2 and LAK cell therapy.

| | | *No. Patients* | *Responses* |
|---|---|---|---|
| Pulse | Extramural NCI IL-2+LAK | 64 | 2 CR; 9 PR (17%) |
| Infusion | Biotherapeutics IL-2+LAK | 40 | 0 CR; 13 PR (33%) |

CR = complete response; PR = partial response.

The Rosenberg approach did produce severe toxicity (Rosenberg *et al.*, 1985, 1987), all patients needing intensive care and occasionally ventilation. Though there were treatment related deaths initially, in contrast to chemotherapy the majority began to recover within 2–3 h of stopping IL-2 and most were fit enough to return home 24–48 h after stopping treatment without long term sequelae.

*Toxicity of high dose IL-2 + LAK cells*

The list of side effects seen in these patients is very extensive (Table III), though it is not clear how much is a direct effect of IL-2 and how much an effect of other biological factors such as gamma interferon known to be induced by IL-2 (Lotze *et al.*, 1985).

The principal toxicity is fluid retention and capillary leakage (Lotze *et al.*, 1987; Rosenstein *et al.*, 1986) which manifest as weight gain and oedema, hypotension, oliguria or anuria and in some patients hepatic pulmonary failure and even coma if treatment is continued at high dosage (Margolin *et al.*, 1987). An interesting aspect of the toxicity associated with the use of high dose IL-2 was erythrodermia with severe pruritis occassionally producing a clinical picture similar to that of the Mazzotti syndrome seen in patients with micro filarial skin disease on treatment with diethyl carbimazine (Wilcockes & Manson-Bahr, 1972).

This observation, which could be due to an autoallergic reaction against skin or a skin commensal organism, taken together with the anecdotal reports of autoimmune arthralgias and thyroiditis in patients receiving IL-2 raises the question as to whether this lymphokine might induce autoimmunity as well as produce an antitumour response. This is an interesting paradox as it is now more than 25 years since Macfarlane Burnett first speculated (Burnet, 1969; Burnet, 1976) that autoimmunity might be the extreme manifestation of the body's mechasnism for counteracting attempts by malignant tumours to escape from immune surveillance. As gamma interferon is known to be induced by IL-2 (Lotze *et al.*, 1985) these side effects could be due to aberrant induction of class II MHC antigen as has been speculated for autoimmune disease occurring after viral infection (Bottazzo *et al.*, 1983). Despite this impressive list of toxicities, as they were greatly diminished in patients on steroids (Vetto *et al.*, 1987), steroids provide a safety net if severe toxicity occurs, though as they also eliminate the therapeutic effect of IL-2 they could not be used prophylactically. This and the observation that all of this toxicity disappeared more rapidly than those after chemotherapy, once the drug was stopped, suggests that in the long term toxicity will not be the major factor limiting IL-2 use.

*Therapeutic endeavours attempting to reduce toxicity of IL-2 + LAK cells*

There have been several attempts to find less toxic ways of giving the treatment. Few responses were seen when repeated courses of lower non-toxic doses of IL-2 alone were given over a period of 4 weeks or more (Taguchi & Kimoto, 1986; Bradley *et al.*, 1986) and there was no increase in the number of responses when LAK cells were added to this lowdose IL-2 regimen in the multicentre Japanese study (Taguchi & Kimoto, 1986).

The most significant attempt to reduce toxicity was that of West *et al.*, (1986) who gave the drug by continuous infusion rather than the 8 hourly bolus injection regime used by Rosenberg's group. Studies in animal tumour models had demonstrated that antitumour effect related more to the duration of exposure than to the peak levels achieved

**Table III** Toxicity of IL-2±LAK.

| | |
|---|---|
| FEVER | Controlled by antiprostaglandins |
| GI TRACT & LIVER | Vomiting or diarrhoea ±jaundice |
| CNS | Euphoria, mania, psychosis, coma |
| SKIN | Erythrodemia – pseudo Mazzoti reaction |
| RENAL | Like ATN but more rapidly reversed |
| HAEMATOLOGICAL | Eosinophilia |
| CAPILLARY LEAK | Common but responsive to steroids |
| AUTOIMMUNE DISEASE | Rare and steroid sensitive |

(Cheever *et al.*, 1985). Furthermore, since the acute toxicity of IL-2 is reversed rapidly when treatment is stopped, continuous infusion provided a safer way of regulating dosage and avoiding the crash which usually terminated treatment when it was given by the intensive 8 hourly pulse schedule. In fact using continuous infusion only 6/40 of the patients of West *et al.* (1987) could not be managed on an ordinary ward and the problems were even less with more regular temporary interruption of infusions.

Recently further phase I/II testing of this type of schedule using IL-2 alone has reported response in 9 of 18 patients treated (Paciucci *et al.*, 1988). From this study and that of West *et al.*, there is increasing evidence that the most important marker which correlates with response is level of lymphocyte activation demonstrated *in vivo*, though Cohen *et al.* (1987) have demonstrated that those patients whose tumours demonstrated enhanced HLA-DR expression during IL-2 treatment also showed a higher frequency of response.

*Influence of tumour type on response to IL-2/LAK*

To date most of the phase I/II testing of IL-2/LAK has been done in patients with primarily chemotherapy resistant renal cell carcinoma, melanoma and colon cancer (Table IV) (Rosenberg *et al.*, 1987; West *et al.*, 1987; Fisher *et al.*, 1987; Dutcher *et al.*, 1987).

However, despite less data there are already tantalising anecdotes which suggest that the response rate may be even higher in the chemosensitive tumours such as head and neck (Taguchi & Kimoto, 1986), bladder cancer (Pizza *et al.*, 1984) and relapsed lymphoma (Rosenberg *et al.*, 1987).

*Future approaches to the use of IL-2*

Given that at present all the standard modalities of cancer treatment such as surgery, radiotherapy and chemotherapyare immunosuppressive, IL-2 in combination will undoubtedly be the next direction to explore. Preliminary results from Mitchell (1987) reporting one complete response and five partial responses in 24 patients receiving low dose IL-2 in combination with low dose cyclophosphamide, a drug not known to have appreciable activity in melanoma, provide an early indication of this potential.

A further approach for enhancing the activity of IL-2 comes from animal studies showing more than additive antitumour activity when given in combination with either alpha interferon (Truitt *et al.*, 1987) or tumour necrosis factor, though accurate assessment of therapeutic ratio and precise dosage in these models is difficult. This is particularly so now that there is some evidence for a bell-shaped rather than linear dose response curve to these treatments (Talmadge *et al.*, 1987).

**Table IV** Tumour cell type and response to IL-2+LAK.

| | *No. Patients* | *No. Responses* |
|---|---|---|
| Renal cell | 74 | 6 CR; 14 PR (27%) |
| Melanoma | 68 | 2 CR; 15 PR (25%) |
| Colon | 39 | 1 CR; 2 PR (8%) |

CR = complete response; PR = partial response.

The future potential of activated cell therapy remains more uncertain particularly because of their cost and logistical problems. One of the most exciting laboratory developments has been the demonstration by Rosenberg's group in experimental animal tumours that tumour infiltrating lymphocytes (Yron *et al.*, 1980; Rosenberg *et al.*, 1986) (TIL) can be grown out of tumour cell suspensions under the influence of IL-2 and are 100-fold more active than LAK cells in adoptive immunotherapy models (Rosenberg *et al.*, 1986) and can enhance the activity of radiotherapy and chemotherapy. Since this original publication there have been multiple studies (Whiteside *et al.*, 1987; Von Flidener *et al.*, 1987; Belldegrum & Rosenberg, 1987) showing that it is possible to get similar cells from a large proportion of human tumours.

Although monoclonal antibodies as therapeutic agents have been less investigated than IL-2, there have been two recent innovations suggesting that combinations of IL-2 activated cells and monoclonal antibodies may provide a more immunologically specific treatment in the future.

Firstly, pretreatment of peripheral blood buffy coat cells with anti T3 antibody prior to IL-2 activation (Ochoa *et al.*, 1987) was shown to enhance the generation of LAK cells and Lotz *et al.* (1987) have demonstrated that cytotoxicity was further enhanced if an anti T3 antibody was crosslinked to a tumour specific antitumour antibody. To date there were no clinical data to support this approach though the preliminary results of Douillard *et al.* (1986) reporting 1 complete and 4 'partial' responses in 20 patients with metastatic colon cancer treated with monoclonal antibody bound to peripheral blood buffy coat cells does suggest that this may be a way to make LAK cell therapy more immunologically specific.

In conclusion, compared with the small adjuvant studies which initiated the interest in BCG plus allogeneic tumour cell immunotherapy and interferon, the data reviewed suggest that interleukin-2 has a more secure foundation as a treatment worth further exploration.

Although to date only chemo-insensitive tumours such as renal cell carcinoma, melanoma, colon and lung cancer have been adequately screened, the preliminary results in chemotherapy resistant lymphoma, in head and neck and bladder cancer suggests that the use of IL-2 may not be limited to just those tumours which have been tested to date.

The expense and complexity of the IL-2/LAK cell programme combined with its excessive toxicity make it important to clarify the need for LAK cell therapy as continuous infusion may be a simpler and safer way to give the IL-2, and have greater potential clinically.

For the future, the preliminary data from combining IL-2 with chemotherapy and those from combining TIL with both chemotherapy and radiotherapy are most encouraging given the known immunosuppressive effect of all three principal cancer treatment modalities. These results and those demonstrating synergism between IL-2 and alpha interferon or tumour necrosis factor and LAK cells coated with monoclonal antibodies suggest that the full potential of this lymphokine has yet to be realised.

### References

ANON. (1985). Review and Outlook: Cancer Patience. *The Wall Street Journal,* December 16th.

BELLDEGRUM, A. & ROSENBERG, S.A. (1987). Antitumour and proliferative responses of IL-2 expanded tumour infiltrating lymphocytes. *Fed. Proc.,* **46,** 1508.

BOTTAZZO, G.F., PUJOL-BORRELL, R., HANAFUSA, T. & FELDMAN, M. (1983). Role of aberrant HLA-DR expression and antigen presentation in induction of endocrine autoimmunity. *Lancet,* **ii,** 1115.

BRADLEY, M., KONRAD, S. & DEGROAT, M. (1986). *In vivo* normalization of NK activity in patients with cancer treated with Interleukin-2. *Proc. Amer. Assoc. Cancer. Res.,* **27,** 1394 (*abstract*).

BURNET, F.M. (1969). *Self and Not-self.* Cambridge University Press, Cambridge, UK.

BURNET, F.M. (1976). *Immunology, Ageing and Cancer.* W.H. Freeman & Co.: Oxford, UK.

BYLINSKY, G. (1985). Science scores a cancer breakthrough. *Fortune Magazine,* November 25, p. 16.

CHASE, M. (1986). Latest findings on cancer drug fall short of dazzling first results. *The Wall Street Journal,* November 12.

CHEEVER, M.A., THOMPSON, J.A., KERN, D.E. & GREENBERG, P.D. (1985). Interleukin 2 (IL2) administered *in vivo*: Influence of IL2 route and timing on T cell growth. *J. Immunol.,* **134,** 3895.

COHEN, P.J., LOTZE, M.T., ROBERTS, J.R., ROSENBERG, S.A. & JAFFE, E.S. (1987). Immunopathology of sequential tumour biopsies in patients on IL-2. *Am. J. Pathol.,* **129,** 208.

DELORME, E.J. & ALEXANDER, P. (1964). Treatment of primary fibrosarcoma in the rat with immune lymphocytes. *Lancet,* **ii,** 117.

DOUILLARD, J.Y., LE MEVEL, B., CURTET, C., VIGNOUD, J., CHAPAL, J.F. & KOPROWSKI, H. (1986). Immunotherapy of gastrointestinal cancer with monoclonal antibodies. *Med. Oncol. Tumor Pharmacother.,* **3,** 141.

DUTCHER, J.P., CREEKMORE, S., WEISS, G.R. & 4 others (1987). Phase II study of IL-2/LAK in patients with melanoma. *Proc. Amer. Assoc. Clin. Oncol.,* **6,** 246 (*abst. 790*).

EINHORN, L.H. & DONOGHUE, J.P. (1977). Cis-diaminedichloroplatinum, vinblastine and bleomycin combination chemotherapy in disseminated testicular cancer. *Ann. Int. Med.,* **7,** 293.

FISHER, R.I., COLTMAN, C.A., DOROSHOW, J.H. & 4 others (1987). Phase II clinical trial of IL-2 plus LAK in metastatic renal cell cancer. *Proc. Amer. Soc. Clin. Oncol.,* **6,** 244 (*abst. 959*).

GILLIS, S. & WATSON, J. (1980). Biochemical and biological characterization of lymphocyte regulatory molecules. V. Identification of an Interleukin-2 producing human leukemia T cell line. *J. Exp. Med.,* **152,** 1709.

GRESSER, I. & BOURALI, C. (1970). Antitumour effects of interferon preparations in mice. *J. Natl Cancer Inst.,* **45,** 365.

GRESSER, I., BROUTY-BOYE, D., THOMAS, M.-T. & MACIEIRA-COELHO, A. (1970). Interferon and cell division, 1. Inhibition of the multiplication of mouse leukemia L 1210 cells *in vitro* by interferon preparation. *Proc. Natl Acad. Sci.,* **66,** 1052.

GRIMM, E.A., MAZUMDER, A., ZHANG, H.Z. & ROSENBERG, S.A. (1982). The lymphokine activated killer cell phenomenon: Lysis of NK resistant fresh solid tumour cells by IL-2 activated autologous human peripheral blood lymphocytes. *J. Exp. Med.,* **155,** 1823.

GROSSMAN, Z. & HERBERMAN, R.B. (1986). Natural killer cells and their relationship to T-cells: Hypothesis on the role of T-cell receptor gene rearrangement on the course of adaptive differentiation. *Perspect. Cancer Res.,* **46,** 2651.

HAVELE, C., BLEACKLEY, R.C. & PAETKAU, V. (1986). Conversion of specific to nonspecific cytotoxic T lymphocytes. *J. Immunol.,* **137,** 1448.

HIGBY, D.J., WALLACE, H.J., ALBERT, D.J. & HOLLAND, J.F. (1974). Diaminadichloroplatinum: A phase I study. *Cancer,* **33,** 1219.

KIRKWOOD, J.M. & ERNSTOFF, M. S. (1984). Interferons in the treatment of human cancer. *J. Clin. Oncol.,* **2,** 336.

KROWN, S.E. (1986). Interferons and Interferon inducers in cancer treatment. *Semin. Oncol.,* **13,** 207.

LAFRENIERE, R. & ROSENBERG, S.A. (1985). Successful immunotherapy of murine experimental hepatic metastases with lymphokine-activated killer cells and recombinant Interleukin-2. *Cancer Res.,* **45,** 3735.

LARSSON, E.L. (1986). Interleukin-2 and its receptor. *Med. Oncol. Tumour Pharmacother.,* **2,** 231.

LEE, S.K. & OLIVER, R.T.D. (1978). Autologous leukemia-specific T-cell-mediated lymphocytotoxicity in patients with acute myelogenous leukaemia. *J. Exp. Med.,* **147,** 912.

LIU, C.C., PERUSSIA, B., COHN, Z.A. & YOUNG, J.D.E. (1986). Identification and characterization of a pore-forming protein of human peripheral blood NK cells. *J. Exp. Med.,* **164,** 2061.

LOTZE, M.T., MATORY, Y.L., ETTINGHAUSEN, S.E. & 5 others (1985). *In vivo* administration of purified human Interleukin 2. II. Half life, immunologic effects, and expansion of peripheral lymphoid cells *in vivo* with recombinant IL2. *J. Immunol.,* **135,** 2865.

LOTZE, M.T., MATORY, Y.L., RAYNER, A.A. & 4 others (1986). Clinical effects and toxicity of Interleukin-2 in patients with cancer. *Cancer,* **58,** 2764.

LOTZE, M.T., ROBERTS, K., CUSTER, M.C., SEGAL, D.A. & ROSENBERG, S.A. (1987). Specific binding and lysis of human melanoma by IL-2 activated cells coated with Anti-T3 or Anti-Fc receptor crosslinked to anti-melanoma antibody: A possible approach to the immunotherapy of human tumours. *J. Surg. Res.* **42,** 580.

MARGOLIN, K., JAFFE, H.S., HAWKINS, M. & 4 others (1987). Toxicity of IL-2/LAK cell therapy. *Proc. Amer. Soc. Clin. Oncol.,* **6,** 251 (*abst. 988*).

MATHE, G., AMIEL, J.L., SCHWARZENBERG, L. & 5 others (1969). Active immunotherapy for acute lymphoblastic leukaemia. *Lancet,* **i,** 697.

MATHE, G., POUILLART, P. & LAPEYRAQUE, F. (1969). Active immunotherapy of L1210 leukemia applied after the graft of tumour cells. *Br. J. Cancer,* **23,** 814.

MAZUMDER, A., EBERLEIN, T.J., GRIMM, E.A. & 4 others (1984). Phase I study of the adoptive immunotherapy of human cancer with lectin activated autologous mononuclear cells. *Cancer,* **53,** 896.

MAZUMDER, A. & ROSENBERG, S.A. (1984). Successful immunotherapy of NK-resistant established pulmonary melanoma metastases by the intravenous adoptive transfer of syngeneic lymphocytes activated *in vitro* by Interleukin-2. *J. Exp. Med.,* **159,** 495.

MITCHISON, N.A. (1953). Passive transfer of transplantation immunity. *Nature,* **171,** 267.

MITCHELL, M.S. (1987). Effectiveness and tolerability of low dose cyclophosphamide and Interleukin-2 in disseminated malignant melanoma. *Amer. Soc. Clin. Oncol. Education Booklet,* p. 17.

MORGAN, D.A., RUSCETTI, F.W. & GALLO, R. (1976). Selective *in vitro* growth of T lymphocytes from normal human bone marrows. *Science,* **193,** 1007.

MORTEL, C.G. (1986). On lymphokines, cytokines and breakthroughs. *J. Am. Med. Assoc.,* **256,** 3141.

MULE, J.J., SHU, S., SCHWARZ, S.L. & ROSENBERG, S.A. (1984). Adoptive immunotherapy of established pulmonary metastases with LAK cells and recombinant Interleukin-2. *Science,* **225,** 1487.

MUUL, L.M., DIRECTOR, E.P., HYATT, C.L. & ROSENBERG, S.A. (1986). Large scale production of human lymphokine activated killer cells for use in adoptive immunotherapy. *J. Immun. Meth.,* **88,** 265.

OCHOA, A.C., GROMO, G., ALTER, B.J., SONDEL, P.M. & FRITE, H.B. (1987). Long-term growth of lymphokine killer (LAK) cells: Role of anti-CD3, beta-IL1, interferon-gamma and -beta. *J. Immunol.,* **138,** 2728.

OLIVER, R.T.D. (1982). Biology of host/tumour cell interaction. In *Scientific Foundations of Urology* (2nd ed), Chisholm, G.D. & Williams, D.I. (eds), p. 624. Wm. Heinemann: London, UK.

OLIVER, R.T.D. (1986). Rare cancers and specialist centres. *Br. Med. J.,* **292,** 641.

OLIVER, R.T.D. & LEE, S.K. (1979*a*). Self-restricted cytotoxicity against acute myeloid leukemia cells. In *Natural and Induced Cell-Mediated Cytotoxicity,* Riethmuller *et al.* (eds) p. 183. Academic Press: New York.

OLIVER, R.T.D. & LEE, S.K. (1979*b*). Histocompatibility antigens and T cell responses to leukemia antigens. In *Modern Trends in Human Leukemia* III, Neth *et al.* (eds), p. 377. Springer-Verlag: Berlin.

ORTALDO, J.R. & HERBERMAN, R.B. (1984). Heterogeneity of natural killer cells. *Ann. Rev. Immunol.,* **2,** 359.

PACIUCCI, P.A., BARDWAJ, S., ODCHIMAR, R., GLIDEWELL, O. & HOLLAND, J.F. (1988). Immunotherapy for metastatic cancer with recombinant IL-2 by continuous infusion. *Proc. Amer. Soc. Clin. Oncol.,* **7,** 163 (abstr. 630).

PINSKY, C.M., CAMACHO, F.J., KERR, D., GELLER, N.L., KLEIN, F.A., HERR, H.A., WHITMORE, W.F. Jr. & OETTGEN, H.F. (1985). Intravesical administration of Bacillus Calmette-Guerin in patients with recurrent superficial carcinoma of the urinary bladder: Report of a prospective, randomized trial. *Cancer Treat. Rep.*, **69**, 47.

PIZZA, G., SEVERINI, G., MENNITI, D., DEVINCI, C. & CORRADO, F. (1984). Tumour regression after intralesional injection of Interleukin 2 (IL-2) in bladder cancer. Preliminary report. *Int. J. Cancer*, **34**, 359.

POWLES, R.L., RUSSELL, J., LISTER, T.A., OLIVER, R.T.D., HAMILTON-FAIRLEY, G. & ALEXANDER, P. (1977). Immunotherapy for AML. *Br. J. Cancer*, **35**, 265.

RAYNER, A.A., GRIMM, E.A., LOTZE, M.T., CHU, E.W. & ROSENBERG, S.A. (1985). Lymphokine-activated killer (LAK) cells. Analysis of factors relevant to the immunotherapy of human cancer. *Cancer*, **55**, 1327.

ROSENBERG, S.A., LOTZE, M.T., MUUL, L.M. & 10 others (1985). Observations on the systemic administration of autologous lymphokine-activated killer cells and recombinant Interleukin-2 to patients with metastatic cancer. *N. Engl. J. Med.*, **313**, 1485.

ROSENBERG, S.A., LOTZE, M.T., MUUL, L.M. & 10 others (1987). A progress report on the treatment of 157 patients with advanced cancer using lymphokine-activated killer cells and Interleukin-2 or high-dose Interleukin-2 alone. *N. Engl. J. Med.*, **316**, 898.

ROSENBERG, S.A., SPIESS, P. & LAFRENIERE, R. (1986). A new approach to the adoptive immunotherapy of cancer with tumor-infiltrating lymphocytes. *Science*, **233**, 1318.

ROSENBERG, S.A. & TERRY, W. (1977). Passive immunotherapy of cancer in animals and man. *Adv. Cancer Res.*, **25**, 323.

ROSENSTEIN, M., EBERLEIN, T.J. & ROSENBERG, S.A. (1984). Adoptive immunotherapy of established syngeneic solid tumors. Role of T lymphoid subpopulations. *J. Immunol.*, **132**, 2117.

ROSENSTEIN, M., ETTINGHAUSEN, S.E. & ROSENBERG, S.A. (1986). Extraversation of intravascular fluid mediated by the systemic administration of recombinant Interleukin-2. *J. Immunol.*, **137**, 1735.

SCHMIDT, R.E., BARTLEY, G.T., LEE, S.S. & 6 others (1986). Expression of the NKTa clonotype in a series of human natural killer clones with identical cytotoxic specificity. *J. Exp. Med.*, **163**, 812.

SCHMIDT, R.T., MURRAY, C., DALEY, J.F., SCHLOSSMAN, S.F. & RITZ, S. (1986). A subset of natural killer cells in peripheral blood displays a mature T cell phenotype. *J. Exp. Med.*, **164**, 351.

SLOVIN, S.F., LACKMAN, R.D., FERRONE, S., KIELY, P.E. & MASTRANGELO, M.J. (1986). Cellular immune response to human sarcomas: Cytotoxic T cell clones reactive with autologous sarcomas. *J. Immunol.*, **137**, 3042.

SMITH, K.A. (1980). Continuous cytotoxic T-cell lines. In *Contemporary Topics in Immunobiology*, Warner, N.L. (ed) **11**, p. 139. Plenum Press, New York.

STRANDER, H., CANTELL, K., INGIMARSON, S., JAKOBSSON, P.A., MILSSONE, V. & SODERBERG, G. (1974). Interferon treatment of ostogenic sarcoma: A clinical trial. *Conference on Modulation of Host Immune Resistance in the Prevention of Treatment of Induced Neoplasias*. Washington DC., U.S. Government Printing Office, **28**, 377.

TAGUCHI, T. & KIMOTO, I. (1986). Interleukin 2 & cancer therapy. *Oncologia*, **18**, 71 and *Proc. 2nd Nagoya International Symposium on Cancer Treatment*.

TALMADGE, J.E., TRIBBLE, H., PHILLIPS, H., TRIBBLE, H.E. & PENNINGTON, R. (1987). Combination chemoimmunotherapy and immunotherapy for metastatic disease. *Proc. Amer. Assoc. Cancer Res.*, **28**, 399 (*abstr. 1582*).

TANIGUCHI, T., MATSUI, H., FUJITA, T. & 4 others (1983). Structure and expression of a cloned cDNA for human Interleukin-2. *Nature,* **302**, 305.

TANIGUCHI, T., MATASUI, H., FUJITA, T. & 6 others. (1986). Molecular analysis of the Interleukin-2 system. *Immunol. Rev.*, **92**, 121.

THURMAN, G.B., MALUISH, A.E., ROSSIO, J.L. & 10 others (1986). Comparative evaluation of multiple lymphoid and recombinant human Interleukin-2 preparations. *J. Biol. Response Mod.*, **5**, 85.

TOUFEXIS, A. & JUCIUS, A. (1980). Interferon: The If drug for cancer. *Time Magazine*, **115**, 38.

TRUITT, G.A., STERN, L.L. & BONTEMPO, J. (1987). Recombinant IL-2 alone and in combination with Interferon alpha. *Proc. Amer. Assoc. Cancer Res.*, **28**, 369 (*abstr. 1463*).

VANKY, F., ROBERTS, T., KLEIN, E. & WILLEMS, J. (1987). Autotumour immunity in patients with solid tumours: Participation of CD3 complex and MHC Class I antigens. *Immunol. Lett.*, **16**, part 1, 21.

VETTO, J.T., PAPA, M.Z., LOTZE, M.T., CHANG, A.E. & ROSENBERG, S.A. (1987). Reduction of toxicity of IL-2/LAK cells in humans by administration of corticosteroids. *J. Clin. Oncol.*, **5**, 496.

von FLIDENER, V., QIAO, L., WHITESIDE, T.L., LEYVRAZ, S., BARRAS, C. & MIESCHER, S. (1987). Clonogenic and functional potential of human tumour infiltrating T lymphocytes. In *Cellular Immunotherapy of Cancer*, Truitt, R.L., Gale, R.P. & Bortin, M.M. (eds) p. 223, Alan R. Liss: New York.

VOSE, B.M., VANKY, F., FOPP, M. & KLEIN, E. (1978). Restricted autologous lymphocyte toxicity in lung neoplasia. *Br. J. Cancer*, **38**, 375.

WAGNER, H., HARDT, C., HEEG, K., ROLLINGHOFF, M. & PFIZENMAIER, K. (1980). T cell derived helper factor allows *in vivo* induction of cytoxic T cells in nu/nu mice. *Nature*, **284**, 278.

WEST, W.H., TAUER, K.W., YANNELLI, J.R. & 4 others. (1987). Constant-infusion recombinant interleukin-2 in adoptive immunotherapy of advanced cancer. *N. Engl. J. Med.*, **136**, 898.

WHITESIDE, T.L., HEO, D.S., CHEN, K., ADLER, A., JOHNSON, J.T. & HERBERMAN, R.B. (1987). Expansion of tumour infiltrating lymphocytes from human solid tumours using IL-2. In *Cellular Immunotherapy of Cancer*, Truitt, R.L., Gale, R.P. & Bortin, M.M. (eds) p. 213. Alan R. Liss: New York.

WILCOCKES, C. & MANSON-BAHR, PEC (1972). Filariases. In *Manson's Tropical Diseases*, p. 233. Bailliere & Tindall: London, UK.

WOODRUFF, M.F.A., SYMES, M.O. & ANDERSON, N.F. (1963). The effect of intraperitoneal injection of thoracic duct lymphocytes from normal and immunised rats in mice innoculated with the Landschutz ascites tumour. *Br. J. Cancer*, **17**, 482.

YANNELLI, J.R., SULLIVAN, J.A., MANDELL, G.L. & ENGLEHARD, V.H. (1986). Reorientation and fusion of cytotoxic T lymphocyte granules after interaction with target cells as determined by high resolution cinemicrography. *J. Immunol.*, **136**, 377.

YRON, I., WOOD, T.A., SPIESS, P.J. & ROSENBERG, S.A. (1980). *In vitro* growth of murine T cells: V. The isolation and growth of lymphoid cells infiltrating syngeneic solid tumors. *J. Immunol.*, **125**, 238.